# The Effect of a Multi-Ingredient Pre-Workout Supplement on Time to Fatigue in NCAA Division I Cross-Country Athletes

**DOI:** 10.3390/nu13061823

**Published:** 2021-05-27

**Authors:** Haley Fye, Caroline Pass, Kate Dickman, Eric Bredahl, Joan Eckerson, Jacob Siedlik

**Affiliations:** Department of Exercise Science and Pre-Health Professions, Creighton University, Omaha, NE 68178, USA; haley-fye@uiowa.edu (H.F.); carolinepass@creighton.edu (C.P.); katedickman@creighton.edu (K.D.); ericbredahl@creighton.edu (E.B.); joaneckerson@creighton.edu (J.E.)

**Keywords:** ergogenic aid, sports nutrition, running performance, caffeine, multi-ingredient pre-workout supplement, endurance, acute

## Abstract

This investigation aimed to determine the effect of a multi-ingredient pre-workout supplement (MIPS) on heart rate (HR), perceived exertion (RPE), lactate concentration, and time to fatigue (TTF) during a running task to volitional exhaustion. Eleven NCAA Division I cross-country runners (20 ± 2 year; height: 171 ± 14 cm; weight: 63.5 ± 9.1 kg) participated in this randomized, double-blind, placebo-controlled cross-over study. Bayesian statistical methods were utilized, and parameter estimates were interpreted as statistically significant if the 95% highest-density intervals (HDIs) did not include zero. TTF was increased in the MIPS condition with a posterior Mean_diff_ = 154 ± 4.2 s (95% HDI: −167, 465) and a 0.84 posterior probability that the supplement would increase TTF relative to PL. Blood lactate concentration immediately post-exercise was also higher in the MIPS condition compared to PL with an estimated posterior Mean_diff_ = 3.99 ± 2.1 mmol (95% HDI: −0.16, 7.68). There were no differences in HR or RPE between trials. These findings suggest that a MIPS ingested prior to sustained running at lactate threshold has an 84% chance of increasing TTF in highly trained runners and may allow athletes to handle a higher level of circulating lactate before reaching exhaustion.

## 1. Introduction

Multi-ingredient pre-workout supplements (MIPSs) are a subcategory of sports nutrition products formulated to enhance metabolic, physiological, and psychological function during exercise. They have gained widespread popularity among both recreational and competitive athletes. As their name implies, MIPSs contain a blend of ingredients, such as caffeine (CAF), beta-alanine (BA), creatine, taurine, nitric oxide agents, and amino acids, as well as herbs and botanicals that are purported to improve exercise training and performance.

Most studies examining the effectiveness of MIPSs have focused upon measures of strength and power (e.g., one-repetition maximum (1RM), resistance training volume, repetitions to failure, vertical jump) [1,2,3,4,5,6], indices of anaerobic exercise performance (e.g., Wingate test, anaerobic running capacity, critical velocity) [7,8,9,10], as well as their effect on subjective mood, energy, and focus [6,7,9,11,12]. The collective findings suggest that MIPSs are more effective at improving muscular endurance compared to maximal effort anaerobic performance [13]. A few studies [4,14,15,16,17] have examined the effectiveness of MIPSs on endurance exercise performance, but have reported conflicting results.

Two studies by Byars [14,15] reported that the pre-workout sports drink EMPACT™ (Mannatech, Inc., Flower Mound, TX, USA) significantly increased maximal oxygen consumption (VO_2max_) and time to fatigue (TTF) compared to a placebo (PL) following a graded exercise test on a treadmill with college-age recreational athletes. In their initial study, VO_2max_ and TTF were improved by 15.5% and 8.7%, respectively. Similarly, Walsh [17] found that a pre-workout energy drink ingested ten minutes before treadmill exercise at 70% VO_2max_ significantly increased TTF (12.5%) and improved subjective feelings of focus, energy, and fatigue in 15 recreational athletes. In contrast, Lutsch [16] reported that a MIPS ingested 30 min before a 5 km race had no significant effect on race time or subjective feelings of fatigue compared to PL in 20 recreationally trained athletes. Collins [4] also reported that short-term ingestion of a MIPS provided some ergogenic effect on recovery from resistance exercise but had no impact on a 4 km cycling time-trial performance in resistance-trained men and women. The studies mentioned above did not investigate competitive or highly trained endurance athletes, which may contribute to the contrasting results, indicating a need to further investigate MIPSs’ ergogenic effect on endurance performance.

PerformElite™ (EndurElite™, Spearfish, SD, USA) (MIPS-PE) is a relatively new MIPS product specifically formulated for endurance athletes. Like other MIPSs, it contains CAF, BA, taurine, and beetroot, as well as a proprietary blend of mushrooms (PeakO_2_™) and apple and ancient peat polyphenols (elevATP^®^). Together, these ingredients are purported to enhance energy, buffer lactic acid, improve mental focus, reduce perceived effort, and delay the onset of fatigue. To our knowledge, no studies have examined the effect of MIPSs on running performance in highly trained endurance athletes. Therefore, the purpose of the current study was to examine the effect of MIPS-PE on: TTF, heart rate (HR), rating of perceived exertion (RPE), and blood lactate concentrations in elite cross-country athletes. It was hypothesized that MIPS-PE would delay the onset of fatigue and result in lower RPE and lactate concentrations compared to a PL control.

## 2. Materials and Methods

### 2.1. Subjects

Eleven NCAA Division I cross-country athletes (male = 6; age: 20 ± 2 year; height: 177.4 ± 15 cm; weight: 67.5 ± 7.6 kg, female = 5; age: 20 ± 2 year; height: 163.6 ± 7.3 cm; weight: 58.7 ± 9.0 kg) agreed to participate in the study. Inclusion criteria for study enrollment required participants to be active members of the cross-country team. Exclusion criteria were defined as having known cardiovascular, pulmonary, or metabolic disease. All enrolled participants completed the study. Prior to data collection, all participants provided written informed consent and completed a health history questionnaire before participation. The study conformed to the Declaration of Helsinki standards, and the procedures followed were in accordance with the protocol approved by the Institutional Review Board (1471556-1).

### 2.2. Study Protocol

All subjects had completed their in-season competition three days before the first experimental trial, and therefore were at similar training status and peak fitness. None of the subjects had ingested MIPS-PE or any other dietary supplements for a minimum of six weeks before testing, nor were they taking any medications that would affect the study’s outcome. To control for potentially confounding variables, the testing sessions were conducted at the same time of day, with each trial separated by one week. During the study, the subjects were asked to maintain their normal dietary patterns and abstain from other nutritional supplements and nonprescription drugs. Subjects also were asked not to exercise in the 24 h before their testing sessions. Data collection took place over a seven-day period from 18–25 November 2019.

### 2.3. Supplementation

**Figure 1:**
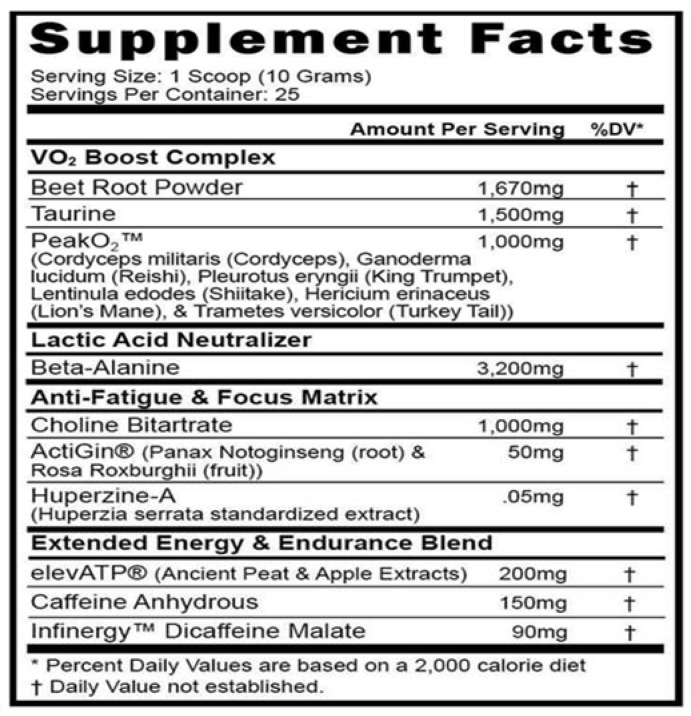
Supplement label information for the PerformElite™.

Thirty minutes before beginning the running protocol, the subjects consumed either MIPS-PE (10 g) or PL (10 g) mixed with 10 oz of water as per manufacturer recommendations. MIPS-PE is a commercially available pre-workout supplement containing beetroot powder, taurine, BA, and a 1000 mg proprietary blend of mushrooms (PeakO_2_^®^, Compound Solutions, Carlsbad, CA, USA) to enhance VO_2_ and buffer lactic acid; an anti-fatigue and focus matrix containing choline bitartrate, a proprietary blend of Panax notoginseng and Rosa roxhurghi fruit (ActiGin^®^; NuLiv Science, Brea, CA, USA), and Huperzine A; and an extended energy and endurance blend containing ancient peat and apple extracts (elevATP^®^, FutureCeuticals, Inc., Momence, IL, USA), CAF anhydrous, and Infinergy™ (Creative Compounds, LLC, Scott City, MO, USA) dicaffeine malate (Figure 1). Potential side effects of the supplement ingredients are outlined in Table S1. The PL consisted of a non-caloric, sugar-free flavored drink (Crystal Light^®^, Kraft Foods, Chicago, IL, USA) similar to MIPS-PE’s taste and appearance. A researcher blinded to the two treatment conditions prepared the drinks prior to testing. Conditions were unblinded after data collection was completed.

### 2.4. Running Protocol

Thirty minutes after consuming their assigned drink, subjects completed a running task to fatigue on a treadmill (Woodway 4Front Treadmill, Woodway, Waukesha, WI, USA) consisting of a 20 min warm-up at each subject’s “easy” pace (Stage 1), followed by a 5 min transition at “marathon” pace (Stage 2), then running to volitional exhaustion at their “threshold” pace (Stage 3), which corresponded to a velocity at or near their lactate threshold (i.e., 80–90% VO_2max_). Paces at each stage were calculated via the Jack Daniels’ Running Formula (JDRF) [18] using each athlete’s record obtained during the last month of the cross-country season [19].

### 2.5. Lactate Testing

Blood sampling for lactate was obtained via finger-stick and measured using a Lactate Scout portable lactate analyzer (EKF Diagnostics, Cardiff, UK) [20]. Resting lactate concentrations were measured after ten minutes of quiet sitting. Lactate was further measured immediately post-exercise, five minutes post-exercise, and ten minutes post-exercise.

### 2.6. Heart Rate and Rating of Perceived Exertion

HR was continuously monitored using a chest-worn heart rate sensor (Polar H10 Heart Rate Sensor, Polar Electro, Bethpage, NY, USA). RPE was measured using the Borg RPE scale (6–20) to obtain a subjective measure of exercise intensity [21]. Both HR and RPE were recorded immediately before starting the running task, every three minutes during the exercise trial, and finally at volitional fatigue immediately after stopping the treadmill.

### 2.7. Statistical Analysis

A power analysis indicated that 11 subjects would exceed 80% power for detecting an effect size of *d* = 0.94 (a large effect as outlined by Cohen [22]) for relevant differences at an alpha < 0.05. Bayesian paired samples *t*-tests analyzed differences in TTF between the two conditions. Heart rate and RPE measures were averaged to provide a representative value for each running protocol stage (1–3). Changes in blood lactate concentration, RPE, and HR were analyzed using Bayesian generalized linear models with Markov chain Monte Carlo (MCMC) estimation. Normality of data was verified using the D’Agostino–Pearson test, and a non-informative, uniform prior was used for all analyses. Reported parameter estimates from Bayesian models include the posterior mean difference and 95% highest-density intervals (HDIs). Parameter estimates were interpreted as statistically significant if the 95% HDI did not include zero. All statistical analyses were performed using R version 3.6.1 [23].

## 3. Results

### 3.1. Time to Fatigue

Time to fatigue was increased in the MIPS-PE condition with an estimated posterior Mean_diff_ = 154 ± 4.2 s (95% HDI: −167, 465) and moderate effect size estimate of 0.46 (95% HDI: −0.48, 1.53). The 95% HDI of the mean difference did include zero, but 84% of the highest-density posterior values were greater than zero, suggesting a strong probability (0.84) that the supplement would generate an increased TTF (Table 1).

### 3.2. Blood Lactate

The lactate concentration immediately post-exercise was higher in the MIPS-PE condition compared to PL, with an estimated posterior Mean_diff_ = 3.99 ± 2.07 mmol (95% HDI: −0.16, 7.68) and a moderate effect size estimate of 0.59 (95% HDI: −0.32, 1.51). There were no meaningful differences in lactate concentrations five and ten minutes post-exercise between trials.

### 3.3. Heart Rate and Rating of Perceived Exertion

There was a significant increase in both HR and RPE across the three exercise stages in both trials (Table 1). However, no significant interactions or main effects were observed between MIPS-PE and PL conditions for either variable.

## 4. Discussion

**Figure 2:**
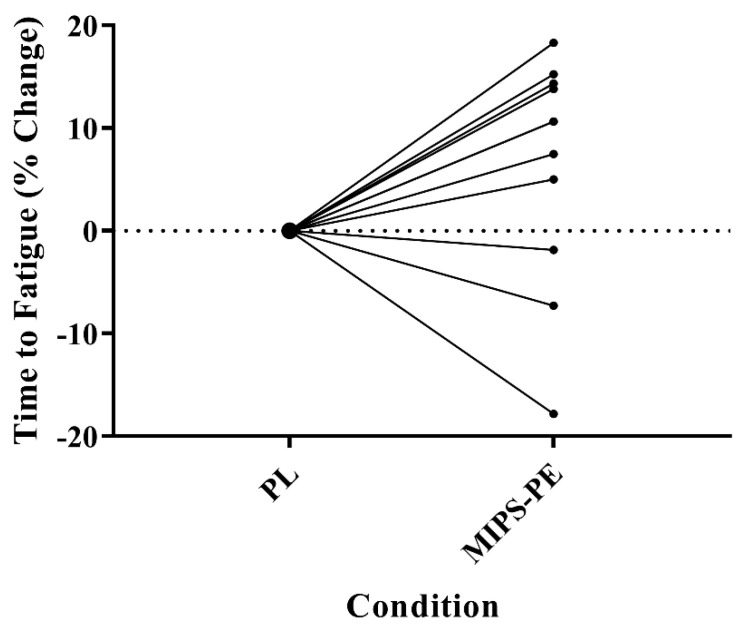
Percent change in time to fatigue in the supplement condition relative to the placebo control. Percent change between the conditions was calculated as follows: [((Supplement/Placebo) − 1) × 100]. Eight of the eleven subjects (72.7%) experienced an increased TTF in the supplement condition relative to the placebo, with an average increase of 6.2 ± 11.0%.

The aim of this project was to quantify the acute effect of a commercially available multi-ingredient pre-workout supplement on a time to fatigue task in Division I cross-country athletes. The current study’s findings suggest that MIPS-PE may increase TTF in highly trained NCAA Division I cross-country athletes compared to a PL-control. The mean percent change in TTF between MIPS-PE and PL conditions was 6.2 ± 11.0%. When examining individual results, 8 of 11 subjects demonstrated improved performance during the MIPS-PE trial (range 5.0–18.3%) (Figure 2). When running at a threshold pace, this supplement potentially increases time to fatigue decoupled from variations in heart rate and perceived exertion. In support of these time to fatigue observations, post-exercise lactate increased following the MIPS-PE condition. Therefore, there is a high probability that this supplement delays fatigue in highly trained cross-country athletes.

Like most MIPS products, MIPS-PE contains a considerable number of different dietary ingredients and more than one proprietary blend (Figure 1), making it difficult to determine which ingredients contribute to changes in performance. Further, investigations on the effect of MIPSs for improved endurance performance are scarce compared to those investigating improvements for strength and power. In a notable correlate to the current study, Walsh et al. [17] examined the effect of a multi-ingredient pre-workout supplement on TTF during treadmill running at 70% VO_2max_ in recreationally active men and women. The results showed that the subjects ran 12.5% longer and reported greater focus, energy, and less fatigue during the supplement trial compared to the PL trial. In contrast, Lutsch et al. [16] used a similar study design and reported that MIPS ingestion had no significant effect on perceived fatigue or 5 km running performance (23.62 ± 2.08 min) compared to PL (23.51 ± 1.97 min) in aerobically trained recreational athletes. Given the ambiguity in the literature, it has been suggested that MIPSs may be more effective for endurance exercise performed at a constant velocity to volitional fatigue versus time trials with a predetermined distance.

Certain ingredients contained in this study’s MIPS-PE likely had no meaningful effect on performance given their relatively small dose amounts (Table 2) for an acute exercise bout, including a proprietary blend of mushrooms, choline bitartrate, Panax notoginseng, and ancient peat and apple extracts. For example, Hirsch [24] reported that one week of supplementation with a mushroom blend that included Cordyceps militaris (4000 mg·day^−1^) had no effect on TTF, VO_2max_, or ventilatory threshold during cycling exercise in 28 recreationally active men and women. Therefore, because the dose of the proprietary mushroom blend included in the MIPS-PE (PeakO_2_™) is 1000 mg, it likely did not affect TTF in the present study. Spector [25] used 20 trained male cyclists to examine the effect of choline bitartrate during supramaximal (*n* = 10; power) and submaximal (*n* = 10) cycling exercise bouts to fatigue at power outputs equivalent to 150% and 70% VO_2max_, respectively. In both exercise conditions, either 2430 mg of choline bitartrate or a PL was ingested one hour before exercise. Neither group reported depleted choline during exercise under the choline bitartrate or PL treatment condition. There were no differences in TTF for either exercise condition, despite increases in serum levels of choline (37% and 52%, respectively) following supplementation with choline bitartrate.

To our knowledge, only two studies have examined the direct effect of elevATP™ on performance. Joy [26] reported that eight weeks of supplementation with elevATP™, in conjunction with a weight-training program, significantly increased lower body strength and improved vertical jump peak velocity compared to PL in resistance-trained males. In another study by Reyes-Izquierdo [27], the authors used a randomized PL-controlled crossover design to examine the effect of an acute dose of elevATP™ on step exercise in sedentary young adults. They reported that subjects took more steps and burned more calories than PL, with no significant differences between treatments for blood glucose or lactate concentrations post-exercise. Although these studies’ findings are compelling, none have evaluated ancient peat and apple extracts’ effects on high-intensity endurance exercise in recreational or trained endurance athletes. In addition, many of the authors associated with the studies described above [26,27,28,29] acknowledged an affiliation with the manufacturer of elevATP™; therefore, more randomized, PL-controlled trials conducted in independent laboratories are necessary to confirm its efficacy for enhancing endurance performance.

The effect of beetroot supplementation has also been studied for its potential ergogenic effects on endurance performance. The recommended dosage for an acute ergogenic effect is 6–12 mmol ingested two to three hours before exercise, with the same amount recommended as a chronic dose over 6–15 days [30,31]. The effectiveness of beetroot for enhancing endurance performance is ambiguous. Some studies report improvements in exercise economy and TTF following both acute and chronic supplementation. In contrast, others report no ergogenic effect despite increased concentrations of nitrate (NO_3_-) and nitrite (NO_2_-) in the circulating plasma. Further, the optimal timing for beetroot ingestion is two to three hours before an acute bout of exercise. Thus, it is unlikely that the beetroot powder contained in the MIPS-PE used in the current study had any meaningful effect on TTF.

Taurine is another common ingredient in MIPSs and has been shown to improve endurance performance when ingested alone or in combination with CAF. For example, Balshaw [32] reported that an acute dose (1000 mg) of taurine ingested two hours before a simulated 3 km race on a treadmill improved time-trial performance by 1.7% in eight trained male middle-distance runners compared to PL. The mechanism of action for the improved performance is unclear, but the authors suggested that taurine may have increased the force production capability of the involved muscles. Because a two hour ingestion period has previously been shown to achieve peak plasma levels of taurine [33], it likely did not affect TTF in the current study.

A recent review and meta-analysis by Ojeda [34] reported a large effect size for distance with BA supplementation (ES = 4.27) and a small effect size for time (ES = 0.25) during TTF tasks performed at intensities in aerobic–anaerobic transition zones (60–100% VO_2max_). Most of the studies included in the review examined the chronic effect of BA supplementation using doses ranging from 2.0 to 6.4 g·day^−1^ for four to ten weeks. However, one study by Ojeda [35] reported that endurance-trained men and women who consumed an acute dose of BA (30 mg·kg^−1^ BW; 1.5 to 2.1 g) 60 min before a running task to fatigue ran 40.5 s longer compared to a PL trial. There were no significant differences between trials for HR, RPE, or lactate concentrations; however, lactate was higher immediately post-exercise during the BA condition (14.80 ± 3.01 mmol·L^−1^) compared to PL (13.84 ± 3.01 mmol·L^−1^), which is similar to this study’s results. The amount of BA contained in the MIPS in the present study is 3200 mg, which equated to a relative dose of 47.4 mg·kg^−1^ BW and 54.5 mg·kg^−1^ BW for the males and females, respectively; therefore, BA may have worked to improve TTF in this experiment.

Caffeine is a key ingredient of MIPS-PE, and it is widely accepted that low-to-moderate CAF doses ranging from 3.0–6.0 mg·kg^−1^ body weight (BW) can improve exercise performance in trained athletes [36]. The total CAF content in MIPS-PE was 217 mg (relative doses of specific supplement components are shown in Table 2), which is within the recommended range to elicit an ergogenic effect. Given that we used an acute dose and the short timeframe from ingestion to exercise, it is reasonable that CAF was the primary ingredient responsible for the observed improvement in TTF in the current study. Although we did not control for dietary intake of CAF in this study, a three-day recall of CAF consumption among the subjects was collected before testing and ranged from 0–190 mg·day^−1^. While a study in combat sport athletes has shown a decreased ergogenic effect of CAF following habitual consumption [37], previous studies have reported that the performance effects from acute CAF ingestion are not impacted by habitual CAF consumption during aerobic events [38,39]. Given the modest range of habitual CAF consumption observed in the current study, we do believe that the dietary CAF ingested by our subjects likely had a minimal effect on performance.

In the current study, MIPS-PE increased TTF by ~2.5 min in highly trained runners compared to PL. Based on the discussion above and given the timing and acute dosing strategy used in the current study, the increase in performance was likely explained by the effects of CAF, with a possibility that BA was at a high enough dose to potentiate its effects. Future studies are warranted, using designs that allow for a direct comparison of MIPS-PE against the separate ingredients included in its formulation to identify the components responsible for eliciting an ergogenic effect. In addition, studies are necessary to determine the effects of long-term supplementation of MIPS-PE on performance.

### Limitations of the Study

Studies examining the effect of MIPSs on endurance performance using highly trained athletes are scarce. Our goal of capturing the supplement-induced response in a time to fatigue task in active Division I athletes introduced limitations in the study design resulting from academic and athletic restrictions. This research was conducted without placing dietary or activity restrictions on the subjects prior to data collection. This allowed us to make an accurate assessment of supplement-induced changes in performance within the context of the athletes’ actual training plan, which we believe is the next evolution in supplement-based research designs. We were fortunate in this investigation that the subjects were all at the same point in their training/peaking cycles and have adhered to a relatively standard training protocol for roughly 16 weeks prior to the study. This provides a relatively equal and highly specific trained status that forms the basis for interpreting the data within the context of NCAA Division I aerobic athletes.

This study is also novel in that Bayesian analyses were utilized to better address the variation inherent to human subject designs, specifically as it relates to studies with small sample sizes [40]. The present study identified small effects representative in scale of supplement-induced changes in exercise performance. Bayesian methods were utilized here to illustrate the probability of parameters given the observed data. Specifically, our analyses provide a probability distribution for an estimate, meaning we can assign a probability to our best estimate of the mean difference and the possible values the parameter may take [41]. We believe that Bayesian estimation provides more informative results about the magnitude of parameters and associated variance, which allows for richer inferences to be derived beyond those allowed for by frequentist methods [42]. Moreover, assigning probabilities to expected outcomes will allow for a better translation of research to practice in a sports performance setting.

## 5. Conclusions

In addition to adhering to optimal dietary strategies, many elite endurance athletes use supplements to enhance their training, performance, and recovery. The current investigation results showed that the ingestion of MIPS-PE effectively increased TTF in highly trained runners at an intensity equivalent to their lactate threshold compared to a PL control. Overall, these data suggest a high probability that acute ingestion of MIPS-PE will improve TTF in Division I cross-country athletes during runs at threshold pace. Given the acute dose and timing of ingestion, the CAF included in the supplement was likely the primary ingredient to explain the ergogenic effect, with a possibility that BA potentiated its effects. We also demonstrate how the use of Bayesian methodology can better inform coaches and practitioners by assigning probabilities to outcomes instead of relying on non-informative, frequentist *p*-values. Future studies using highly trained endurance athletes are warranted to replicate the findings, and should include measures of focus and alertness in addition to RPE, as well as blood and muscle biomarkers that may help to identify the primary ingredients responsible for the ergogenic effect, and should use designs that allow for a direct comparison of the MIPS-PE against its separate constituents.

## Figures and Tables

**Table 1:** Summary data for recorded variables. Data are presented as mean ± SD.

	PL	MIPS-PE
Time to Fatigue (seconds)	2288 ± 354	2413 ± 318
Lactate (mmol)		
At rest	3.7 ± 2.7	3.2 ± 1.6
Immediately post-exercise	13.1 ± 6.8	16.6 ± 4.7
Five minutes post-exercise	7.9 ± 4.1	9.6 ± 2.4
Ten minutes post-exercise	6.9 ± 3.2	7.5 ± 2.4
Average Heart Rate (bpm)		
Stage 1	158 ± 9	156 ± 14
Stage 2	175 ± 8	176 ± 8
Stage 3	185 ± 8	188 ± 7
Average Rating of Perceived Exertion		
Stage 1	9.5 ± 1.4	9.2 ± 1.2
Stage 2	12.9 ± 1.0	12.6 ± 1.1
Stage 3	16.7 ± 1.6	16.6 ± 1.1

PL: Placebo; MIPS-PE: PerformElite multi-ingredient pre-workout supplement.

**Table 2:** Relative doses of supplement components. Data are presented as mean ± SD.

	Average Dose Overall (mg/kg)	Average Male Dose (mg/kg)	Average Female Dose (mg/kg)	Observed Acute Dose for Ergogenic Effect
PeakO2^®^	16.1 ± 2.5	14.9 ± 1.7	17.4 ± 2.9	--
Anti-Fatigue and Focus Matrix	16.9 ± 2.7	15.7 ± 1.8	18.3 ± 3.0	--
elevATP^®^	3.2 ± 0.5	3.0 ± 0.3	3.5 ± 0.6	--
beetroot powder	26.8 ± 4.2	25 ± 2.8	29 ± 4.8	6–12 mmol [30,31]
Taurine	24.1 ± 3.8	22. ± 2.5	26.1 ± 4.4	1000 mg [32]
beta-alanine	51.4 ± 8.1	47.9 ± 5.4	55.6 ± 9.3	30 mg/kg [35]
caffeine	3.5 ± 0.5	3.3 ± 0.4	3.8 ± 0.6	3–6 mg/kg [36]

## Data Availability

The data that support the findings of this study are available on request from the corresponding author, J.S. The data are not publicly available due to the small nature of the data set and the potential that the privacy of the research participants could be compromised.

## Supplementary Materials

The following are available online at https://www.mdpi.com/article/10.3390/nu13061823/s1, Table S1: Potential side effects of ingredients contained in the PerformElite^TM^ supplement.
