# Supplementary material for: The Effect of a Multi-Ingredient Pre-Workout Supplement on Time to Fatigue in NCAA Division I Cross-Country Athletes
Source: Nutrients. 2021 May 27;13(6):1823. doi: 10.3390/nu13061823 (PMC8228073; doi:10.3390/nu13061823)
Supplement: Supplementary file 1 [file nutrients-13-01823-s001.zip › nutrients-1221070-supplementary.pdf]

Table S1: Potential side effects of ingredients contained in the PerformElite™ supplement.

Ingredient	Potential Side Effect	Signs of Toxicity
Beet Root Powder	Pink or red-tinted urine/stools, decrease in blood pressure, spike in blood sugar, high oxalate levels leading to possible kidney stones	N/A
Taurine	Found naturally in meat, dairy, and fish; supplementing with up to 3,000 mg is known to be safe and effective	Acute dose of >3 g
Cordyceps militaris (Cordyceps)	Typical dose is 1,050 mg, increased risk of bleeding in those with bleeding disorders.	N/A
Ganoderma lucidum (Reishi)	Dry mouth, upset stomach, diarrhea, headache, bloody stools	N/A
Pleurotus eryngii (King Trumpet mushroom)	No known side effects to consumption	N/A
Lentinula edodes (Shiitake mushroom)	No known side effects to consumption	N/A
Hericium erinaceus (Lions Mane mushroom)	No known side effects to consumption	N/A
Trametes versicolor (Turkey Tail)	Likely safe for most people when taken by mouth appropriately. For most healthy adults does not cause any side effects. In some people who have received chemotherapy and PSK (extracted from turkey tail) have experienced, nausea, vomiting, low white blood cell counts, and liver problems It is unclear if the side effects were due to the chemotherapy or PSK.	N/A
Beta-Alanine	Paraesthesia ("tingling of the skin"), decrease in taurine levels	N/A
Choline Bitartrate	3.5g is typical and is unlikely to cause side effects, doses above this amount may cause sweating, fishy body odor, diarrhea, or vomiting.	N/A
Panax Notoginseng (root)	Dry mouth, flushed skin, rash, nervousness, sleep problems, headache, nausea, vomiting	N/A
Rosa Roxburghii (fruit)	May cause nausea, vomiting, diarrhea, constipation, heartburn, stomach cramps, fatigue, headache, inability to sleep	N/A
Huperzine-A (huperzia serrata standardized extract)	Nausea, diarrhea, vomiting, dry mouth, constipation, sweating, blurred vision, slurred speech, fainting, restlessness, loss of appetite, contraction, and twitching of muscle fibers, cramping, increased saliva and urine, inability to control urination, high blood pressure, swelling, dizziness, inability to sleep, slowed heart rate	N/A
Ancient Peat	No known side effects to consumption	N/A
Apple Extracts	No known side effects to consumption	N/A
Caffeine Anhydrous	Tolerance to different levels of caffeine depend on body size, typical consumption, medications and genetics. Side effects of heavy caffeine use: Headaches, trouble sleeping, restlessness, anxiety, nausea or lack of appetite, diarrhea	Acute dose of >1 g
Infinergy Dicaffeine Malate	Headache, jitteriness, anxiety, stomach discomfort, nausea, nervousness, and rapid heart rate.	See Caffeine Anhydrous

PerformElite™ Label Warning: Check with a qualified healthcare professional before taking this product. Do not use if you are sensitive to caffeine, pregnant or nursing a baby, under 18 years of age, have any known or suspected medical conditions, and/or if you are taking any prescription of OTC medications. Avoid using with any other caffeinated products.
